# Pupil Fluctuations Signal Intentional Forgetting of Natural Scenes

**DOI:** 10.1111/psyp.70119

**Published:** 2025-08-01

**Authors:** Huiyu Ding, Jonathon Whitlock, Lili Sahakyan

**Affiliations:** ^1^ University of Illinois Urbana‐Champaign Champaign Illinois USA; ^2^ Mississippi State University Starkville Mississippi USA; ^3^ Beckman Institute for Advanced Science and Technology University of Illinois at Urbana‐Champaign Champaign Illinois USA

## Abstract

Studies have revealed that information can be intentionally forgotten when instructed, commonly studied in the laboratory with the directed forgetting (DF) procedure. The current investigation examined pupillometric signals associated with intentional forgetting, as the pupil reflects the activity in the locus coeruleus–norepinephrine (LC‐NE) system that is functionally involved in the neural correlates of intentional forgetting. Experiment 1 employed an item‐method DF paradigm, where participants were presented with natural scenes, each followed by a memory cue to either remember (R) or forget (F) that scene. At test, participants were asked to judge whether the presented scene was the original studied version (i.e., “Old”) or a mirrored variant (i.e., “Lure”). By comparing pupil dilation during test trials between R‐cued and F‐cued scenes for both hit and miss trials, we found greater pupil dilation for F‐cued miss trials compared to R‐cued miss trials, but no difference in pupil dilation between the cue conditions for hit trials. This suggests a unique pupillometric pattern linked to successful intentional forgetting. Experiment 2 was aimed at assessing if memory strength differences could provide an explanation for the observed effect. Instead of DF cues, we manipulated memory strength by repeating a subset of scenes, thereby converting all study items into R‐cued items with different degrees of familiarity. We observed no difference in pupil dilation between strongly encoded and weakly encoded scenes at test, indicating that encoding strength by itself did not explain the difference in pupil dilation resulting from intentional forgetting. Together, these findings provide novel evidence that pupil fluctuations during retrieval index successful intentional forgetting.

## Impact Statement

This study provides novel evidence that pupil dilation serves as a physiological marker of successful intentional forgetting, revealing a distinct pupillometric signature that differentiates active (motivated) from passive (incidental) forgetting. The findings also highlight the involvement of the locus coeruleus–norepinephrine (LC‐NE) system in the neural circuitry underlying active forgetting, as indexed by pupil fluctuations, and contribute to the ongoing theoretical debate regarding the mechanisms of the directed forgetting effect.

## 1 Introduction

Forgetting is often perceived as a flaw of our memory system in part because missing an important meeting or forgetting a recent acquaintance's name can be quite embarrassing. However, given that we encounter an enormous amount of information in our daily lives, forgetting some things can be adaptive, especially if information is unwanted, outdated, wrong, embarrassing, or traumatic. In these instances when the objective is not to remember, forgetting can be the desired outcome. One of the experimental laboratory paradigms that examines how we downregulate unwanted memories is the directed forgetting (DF) procedure invented by Bjork et al. (1968) described in detail below.

The current study aimed to examine forgetting‐related pupil dilations, which closely reflect activity in the locus coeruleus–norepinephrine (LC‐NE) system that was functionally involved in the neural correlates underlying intentional forgetting. We especially emphasize differentiating between *intentional*/*active* forgetting and *incidental*/*passive* forgetting. By intentional/active forgetting, we refer to forgotten memories when one is purposefully aiming to forget (i.e., successful intentional forgetting), whereas by incidental/passive forgetting, we refer to accidentally forgotten memories when the goal was to maintain them in memory (i.e., retrieval failures).

### 1.1 Item‐Method DF Paradigm

In the item‐method DF paradigm, participants are sequentially presented with items, each followed by a memory cue instructing them to either remember (R) or forget (F) that item for an upcoming memory test. Participants are told that only the R‐cued items will appear on the test, while F‐cued items will not, and they are explicitly instructed to attempt to forget the F‐cued items. At test, the memory cues are “canceled” such that all items are included in the recognition test, and participants are asked to endorse any item they remember from the study phase as “old”, regardless of the R/F cue that was associated with that item. The typical result from this procedure is a memory impairment for F‐cued items compared to R‐cued items, known as the directed forgetting (DF) effect. The DF effect has been reliably observed across various memory tests, such as free recall, recognition, cued‐recall, and across a variety of stimuli including words (e.g., Bjork 1970; Muther 1965; MacLeod 1975, MacLeod 1999; Woodward and Bjork 1971), sentences (e.g., Geiselman 1974), and pictorial stimuli (e.g., Ding et al. 2022; Quinlan et al. 2010; Lo et al. 2024). For a recent review of behavioral and neural findings in DF, see Sahakyan (2024), and Werner and Lewis‐Peacock (2025). For a meta‐analysis in DF, see Hall et al. (2021).

Several theoretical accounts have been proposed to explain the emergence of the DF effect. The selective rehearsal account (e.g., Basden et al. 1993; Bjork 1970; MacLeod 1999; Tan et al. 2020) posits that participants selectively rehearse R‐cued items. Specifically, participants maintain an item in working memory until they receive the memory cue, at which point they engage in elaborative and continued rehearsal of that item, whereas the forget cue leads to a termination of rehearsal. Therefore, this account posits that the emergence of the DF effect stems from the passive decay of F‐cued items from working memory due to their lack of rehearsal in response to the forget cue.

Alternatively, the active inhibition accounts posit that additional processes are recruited to selectively suppress the item following the forget cue either through direct inhibition of that item or through unbinding of the item from its context. These active views of the DF effect received support both in behavioral studies (e.g., Fawcett and Taylor 2008, 2010, 2012; Lee et al. 2013; Nickl and Bäuml 2024; Whitlock et al. 2025), fMRI studies (Nowicka et al. 2011; Reber et al. 2002; Rizio and Dennis 2013; Wylie et al. 2008), electrophysiological studies (e.g., Fellner et al. 2020; Hauswald et al. 2011; Hubbard and Sahakyan 2021, 2023; Oehrn et al. 2018), and eye tracking studies (Whitlock et al. 2020). In addition, another active mechanistic explanation for DF comes from the context unbinding account (Chiu et al. 2021), suggesting that the forget instruction actively triggers the separation of the item from its encoding context, resulting in impaired recognition due to a contextual mismatch at retrieval.

### 1.2 Intentional and Incidental Forgetting

Importantly, directed forgetting appears to disrupt memory processes beyond passive forgetting, which further suggests an active process is at play. This notion is supported by empirical evidence demonstrating unique signatures of active forgetting that are distinct from passive/incidental forgetting. For example, Whitlock et al. (2020) monitored eye movements in an item‐method DF procedure in which participants studied object‐scene pairs, each followed by a cue to remember or forget the object. When participants mistakenly forgot R‐cued targets and selected a lure instead, their eye movements were disproportionately drawn to the target, suggesting lingering memory of the target item, whereas viewing of F‐cued targets was reduced, indicating weaker memory traces for actively forgotten targets compared to passively forgotten targets. Moreover, recent behavioral studies using the item‐method DF paradigm with a delayed testing procedure showed that the active form of forgetting accelerates the forgetting rate of information compared to passive forgetting (Nickl and Bäuml 2024; Whitlock, Hubbard, et al. 2024). Importantly, this accelerated forgetting is observed even when initial testing reveals no DF effect due to repetitions, further confirming that information associated with the F‐cue is lost from memory at a faster rate than R‐cued information, even if no differences are observed on an immediate test.

Studies monitoring neurological and electrophysiological signatures of intentional forgetting suggest the recruitment of active inhibitory mechanisms (for a review, see Anderson and Hulbert 2021). For example, in an fMRI study by Rizio and Dennis (2013), back‐sorting the subsequent accuracy on a trial‐by‐trial basis, intentional forgetting (F‐cued miss) was uniquely related to increased activity in the right superior parietal lobe, whereas passive forgetting (R‐cued miss) was related to increased activity in different brain regions that do not overlap with those engaged during intentional forgetting. A similar pattern of dissociation was also reported by Wylie et al. (2008) in another fMRI study of intentional forgetting. Importantly, the authors found a significant negative correlation between activity in the prefrontal cortex and hippocampus, such that increased prefrontal cortex activation predicted reduced hippocampal activity. This negative correlation suggests that inhibition is exerted to downregulate hippocampal representations during intentional forgetting (Rizio and Dennis 2013). Additionally, in an intracranial EEG study, Ten Oever et al. (2021) used encoding‐retrieval similarity (ERS) analysis to index retrieval of specific episodic events. They found that relative to passively forgotten R items, intentionally forgotten F items were associated with more pronounced ERS in the alpha/beta frequency, which is commonly related to functional inhibition.

These findings suggest that a forget instruction does not merely reduce or omit encoding‐related representations but rather actively modifies memory traces, likely through a domain‐general inhibitory mechanism. Behavioral studies comparing performance between DF and stop‐signal task (SST) support this view. For example, longer stop‐signal delay typically leads to greater motor inhibition difficulty (e.g., Logan 1994; Logan and Cowan 1984). Crucially, Hourihan and Taylor (2006) observed that increasing the delay of the Forget instruction in an item‐method DF paradigm enhanced recognition of F‐cued items, reflecting diminished intentional forgetting. Additionally, Hubbard and Sahakyan (2023) presented participants with a stop‐signal task that involved withholding a motor response (i.e., motor inhibition) prior to an item‐method DF procedure. They found that participants with more efficient motor inhibition, as indicated by shorter stop‐signal reaction times, exhibited a greater DF effect. Cross‐task neural decoding analyses revealed that EEG patterns distinguishing successful from unsuccessful response inhibition in the stop‐signal task could also predict successful versus unsuccessful directed forgetting within the same participant in the DF task. It has also been suggested that motor inhibition in SST might be analogous to cognitive suppression in Think‐No‐Think (TNT) paradigm (e.g., Menon et al. 2001; Garavan et al. 2002). Findings from the SST highlight the critical role of the right prefrontal cortex (PFC) in canceling motor actions (e.g., Chevrier et al. 2007; Aron et al. 2004), with beta‐band oscillations in this region linked to successful inhibition (Swann et al. 2009; Wagner et al. 2018). Similarly, studies suggest that motor inhibition in the SST may share mechanistic similarities with cognitive suppression in the TNT paradigm (e.g., Menon et al. 2001; Garavan et al. 2002). For instance, overlapping brain regions (e.g., Anderson and Hulbert 2021; Hanslmayr et al. 2009 ) and ERP components (Logan and Cowan 1984; Mecklinger et al. 2009) implicated in stopping prepotent motor responses also appear during the cognitive inhibition. Converging evidence further indicates that both tasks recruit common neural circuitry (Apšvalka et al. 2022) and rhythmic activity patterns (Castiglione et al. 2019), supporting the notion of a domain‐general inhibitory function (for a review, see Marsh and Anderson 2022).

The findings reviewed above point to a complex neural circuitry underlying intentional forgetting, in which prefrontal regions induced global inhibition on hippocampal (and para‐hippocampal) activity in response to forget instructions. Importantly, this inhibitory mechanism likely interacts with global arousal systems, particularly the locus coeruleus–norepinephrine (LC‐NE) system. The locus coeruleus (LC), a neuro‐modulatory nucleus located in the brainstem, is the primary source of norepinephrine for much of the brain (Berridge and Waterhouse 2003). It plays a central role in regulating arousal (Carter et al. 2010) and is involved in executive function, working memory, and attentional control (e.g., Corbetta et al. 2008; Poe et al. 2020; Sara 2009). Anatomically, the LC receives efferent inputs from the prefrontal cortex (e.g., Arnsten and Goldman‐Rakic 1984; Jodo et al. 1998; Sara 2009) and sends direct projections to the hippocampus, particularly the dentate gyrus (Haring and Davis 1985; Patton and McNaughton 1995; Samuels and Szabadi 2008), forming a functional triad. Disruption of LC activity during learning has been shown to impair memory encoding (Kaufman et al. 2020; Kempadoo et al. 2016; Wagatsuma et al. 2018), further supporting its critical role in memory processes. Given its anatomical connectivity and functional integration with both the prefrontal cortex and hippocampus, the LC is plausibly involved in the mechanisms underlying intentional forgetting. Importantly, LC activity is closely linked to changes in pupil size in both humans and non‐human primates (Joshi et al. 2016; Murphy et al. 2011; see Papesh and Goldinger 2024, for a review). On this basis, the current investigation focused on pupillometric signatures, providing a non‐invasive and temporally sensitive index of LC‐NE involvement in intentional forgetting.

### 1.3 Memory‐Related Pupillometry

In addition to its close relationship to the arousal process and indexing activity of the LC system, pupil dilation has also emerged as a robust physiological marker of long‐term memory processes and was found to be related to both long‐term memory formation and retrieval (for a review, see Kafkas 2024). During encoding, numerous studies found that pupil size changes are predictive of subsequent retrieval success, even though the direction of such correlations remains inconclusive (e.g., Ariel and Castel 2014; Eldar et al. 2016; Gross and Dobbins 2021; Kafkas and Montaldi 2011; Naber et al. 2013; Papesh et al. 2012; Võ et al. 2008; Whitlock, Ding, et al. 2024). During memory retrieval, pupil size changes are larger for a studied item correctly endorsed as old versus an unstudied item that was correctly rejected as new (Heaver and Hutton 2011; Kafkas and Montaldi 2015; Otero et al. 2011; Võ et al. 2008). Pupil size changes also reflect the memory strength of the studied items, evident in greater pupil dilation for items successfully recollected than items judged as familiar (e.g., Otero et al. 2011; Taikh and Bodner 2022), responses subjectively reported with higher confidence (e.g., Papesh et al. 2012; Otero et al. 2011), and deeply encoded compared to shallowly encoded items (Brocher and Graf 2016; Kafkas and Montaldi 2015; Montefinese et al. 2013; Otero et al. 2011, but see Gross and Dobbins 2021). Together, pupil size fluctuations have been found to be closely associated with long‐term memory encoding and retrieval.

Critically, however, this body of work has focused exclusively on studies where participants *intend to remember* information, which is the default assumption of most memory research. That is, pupil sizes were monitored at learning and test when the task demand involved successfully encoding and retrieving the learned information. In contrast, less is known about how pupils would behave when participants *intend to forget* information and, critically, when such intentional forgetting is successful, which is the focus of the current investigation.

To our knowledge, only two prior pupillometric studies have involved the item‐method DF procedure (Scholz and Dutke 2019; Lee 2018). Both studies focused on pupil dilation at the time of encoding (specifically during the R/F memory cue period) and found that pupils dilate more when participants receive the R instruction than the F instruction. Critically, these studies collapsed across correct and incorrect trials, and different patterns might emerge when contrasting conditions within incorrect trials. That is, it could be the case that similar patterns of pupil size differences could be observed on correct trials, similar to observations made in eye‐tracking studies (Whitlock et al. 2020) and fMRI studies (e.g., Rizio and Dennis 2013; Wylie et al. 2008), but diverge on incorrect trials where information is either incidentally or intentionally forgotten, which can be directly tested by examining pupillometric patterns at test.

The current investigation examined the pupillometric signature related to intentional forgetting across two experiments. Experiment 1 used an item‐method DF paradigm involving presenting participants with natural scenes, each followed by an R/F memory cue. At test, participants were instructed to distinguish intact targets from lures which were mirrored flipped versions of target scenes. The critical analysis in Experiment 1 focused on examining pupil fluctuations at test when information was incidentally versus intentionally forgotten. We predicted distinct pupillometric patterns for intentional forgetting compared to incidental forgetting. Specifically, prior research shows that pupil dilations increase with semantic (Kuipers and Thierry 2011; Renner and Włodarczak 2017) and episodic (Siefert et al. 2024) mismatches between visual input and memory representations at retrieval. In DF paradigms, forget cues either alter the item memory representation (Fellner et al. 2020; Ten Oever et al. 2021) or unbind the item‐associated contextual information (Chiu et al. 2021), likely resulting in a mismatch between modified memory representation and its retrieval cue at test. We therefore expected intentionally forgotten items to elicit different pupillary responses than incidentally forgotten ones, though the direction of this effect required empirical testing. Furthermore, we conducted Experiment 2 to further explore the mechanisms underlying the pupillary effect related to intentional forgetting. Specifically, Experiment 2 involved presenting natural scenes during encoding, but varied whether scenes were repeated versus non‐repeated (without the R/F memory cue), in order to contrast pupillometric signatures of intentional forgetting with those relating to strong versus weak memories. Comparing pupillometric patterns across two experiments would help distinguish the mechanism underlying the pupillary effect related to intentional forgetting, specifically disentangling active inhibition and selective rehearsal mechanisms. More details are included in the Experiment 2 section.

### 1.4 Experiment 1

We employed an item‐method DF paradigm using natural scenes as stimuli. Pictorial stimuli such as complex scenes (Hauswald and Kissler 2008), line drawings (Quinlan et al. 2010), images of everyday objects (Scotti and Maxcey 2022), and faces (Ding et al. 2022; Metzger 2011) have typically been found to yield a less pronounced DF effect compared to verbal materials. Studies found that DF impairment only emerged when recognition was probed for visual details of each individual image, but not when participants could simply rely on categorical labels extracted from images (i.e., a picture of a bathroom) (e.g., Ahmad et al. 2019). Additionally, when recognition tests involve highly perceptually similar lures (e.g., plurality‐reversed versions of the targets), a previously absent DF effect with entirely novel lures can emerge with perceptually similar lures (Sahakyan and Foster 2009). Therefore, to maximize the chances of obtaining a DF effect with natural scene images, we used mirrored variants of the originally studied scenes as lures by horizontally flipping the scenes, and participants had to decide whether the scenes were the same or mirrored at test.

## 2 Method

### 2.1 Participants

Participants were 90 undergraduate students from the University of Illinois Urbana‐Champaign who participated in exchange for course credit. The study was approved by the Institutional Review Board of the University of Illinois at Urbana‐Champaign and complied with APA ethical standards in the treatment of participants. All participants gave informed consent prior to inclusion in the study. The sample size was chosen based on previous memory‐related pupillometry research, which typically ranges from 20 to 50^1^. Sensitivity analyses revealed that with 90% power and the current sample size (*N* = 90), we should be able to detect a small‐to‐medium effect (*f* = 0.35). Our sample size therefore is sufficiently powered to detect a large memory‐related pupillometric effect as found in a recent meta‐analysis (*f* = 0.85, Lapteva and Martarelli 2024) and the DF effect (Hall et al. 2021).

### 2.2 Apparatus

Experiments were programmed in E‐prime 2. Eye position was recorded at a rate of 1000 Hz using an Eyelink 1000 desk‐mount eye‐tracking system (SR Research). Prior to the study and the test phase, eye position was calibrated using a 3 × 3 spatial array. Calibration ended with participants fixating on a centrally located cross‐hair, which began each phase. We used an HP LP2065 LCD computer screen, with the resolution set to 1280 × 1024. Participants' chins were placed on a chin rest that prevented any head movements throughout the experiment and was positioned 78.7 cm away from the computer screen.

### 2.3 Stimuli

Stimuli consisted of 40 natural scenes selected from the Fine‐Grained Image Memorability (FIGRIM) dataset (Bylinskii et al. 2015). We made sure that selected scenes were largely asymmetrical so that the mirrored lures were perceptually different enough to be distinct from their original versions. We also presented scrambled boxes of each scene prior to the scene presentation as' luminance masks'. The purpose of these luminance masks was to make sure that the pupil size stabilizes after transitioning from a fixation point on a dark gray screen to a brightly colored display. Doing so ensured that the abrupt brightness change had minimal impact on pupil dilation during the actual scene presentation. The luminance masks were created by scrambling the scene images to remove any scene‐identifying information while retaining low‐level visual properties of the image (Grill‐Spector et al. 1998). Specifically, box scrambling was used, in which each scene image was divided into non‐overlapping 5 × 5 blocks of pixels, and the pixel blocks were then randomly shuffled. Two different luminance masks for each image were used between the study and test phases to avoid any potential association participants might have established between the mask and the actual scene image.

### 2.4 Procedure

**FIGURE 1:**
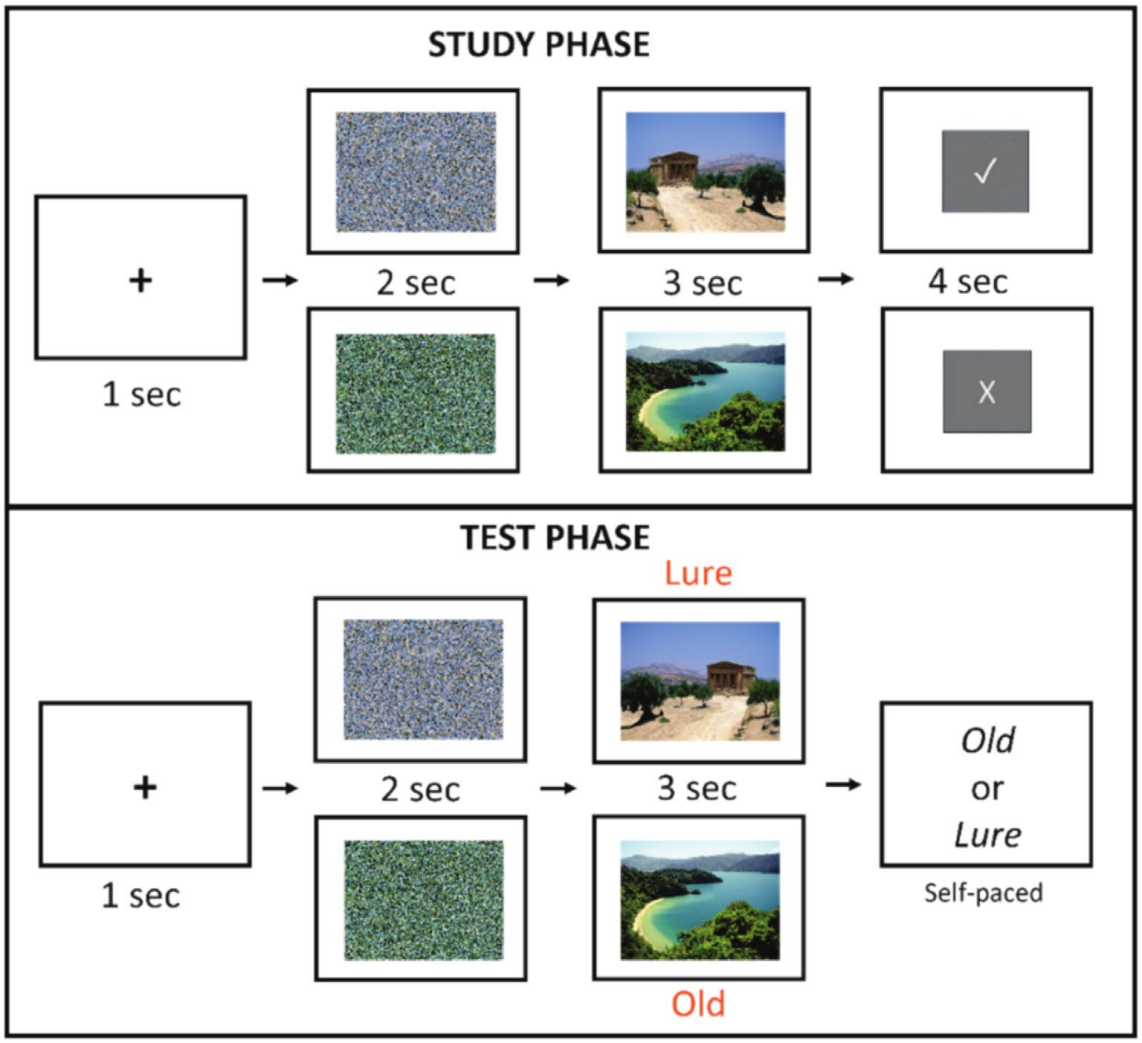
Experimental procedure of Experiment 1. Experiment 1 procedure. Old and Lure labels at test are only shown for illustrative purposes and were not visible to participants during the actual experiment. The check symbol denotes the R cue, and the cross symbol denotes the F cue.

The details of the paradigm are visualized in Figure 1. Participants completed a study phase and a test phase while their eye movements and pupil sizes were monitored throughout the entire procedure. At the beginning of the experiment, participants received instructions and completed practice trials to ensure understanding of the study and test formats. The study phase consisted of 40 trials. Each study trial began with a black fixation cross against a dark gray background presented for 1 s, followed by a luminance mask for 2 s, followed by a natural scene presented for 3 s, and ended with a memory instruction presented for 4 s. Participants were instructed to remember the scene preceding an R cue (a check symbol) because they would appear in the following memory test, and were instructed to forget the scene preceding an F cue (a cross symbol) because they would not be included in the following memory test. The symbols for R and F cues (check and cross) were controlled to have similar luminance values.

Immediately following the study phase, participants received the memory test. Each test trial consisted of a presentation of an initial fixation cross for 1 s, a luminance mask for 2 s, a scene that was either the original studied scene (i.e., Old) or its mirrored variant (i.e., Lure) for 3 s, and finally a response prompt signaling participants to make a response using a button press on a computer keyboard. Participants then made a self‐paced judgment as to whether the scene was Old or Lure. They were instructed to wait for the response prompt before making a button press to minimize eye movements or pupil changes associated with movements in making responses while viewing the scene. The memory test included 20 Old and 20 Lure images, with no overlap (i.e., only one version of the scene was shown at test, either the original or its mirrored version, but not both). The test scenes (Old vs. Lure) and memory cues (R vs. F) were counterbalanced and randomized across conditions.

### 2.5 Analytic Plan

Statistical analyses were conducted using R software (R Development Core Team, 2008). Beta coefficient estimates for fixed and random effects were obtained using the lme4 package (Bates et al. 2015), and significance testing of coefficients was performed using the lmerTest package (Kuznetsova et al. 2017). Follow‐up analyses to significant interactions were performed using the Interactions package (Long 2024) and emmeans package (Lenth 2025). Behavioral results were analyzed with a multilevel SDT approach, by fitting mixed effect regression models with a probit link function to trial‐level binary behavioral accuracy (e.g., Hourihan et al. 2013; Wright et al. 2009). These models produce parameter estimates on the same scale of *d'* in the SDT framework (DeCarlo 1998), with the advantage of simultaneously accounting for item‐and subject‐level variability. Subject‐level discrimination accuracy (*d*' scores) was calculated after hits and false alarms were transformed using a loglinear correction (Hautus 1995; Stanislaw and Todorov 1999) and it was analyzed with paired t‐tests. Subject‐level response bias (*c*) was calculated and analyzed with paired t‐tests across memory conditions. Reaction time data was log‐transformed and analyzed on a trial‐by‐trial basis with a mixed effect model. Outliers with reaction times that were 3 SD away from the mean in reaction time data were removed, accounting for 2% of the data. All models have maximal random effect structures to account for subject‐ and item‐level variability (Barr et al. 2013), including random intercepts for Participants and Items and by‐participant and by‐item random slopes for the fixed effects. Random structures were removed in a stepwise approach if their inclusion resulted in convergence issues. Detailed specifications are reported separately for each model.

### 2.6 Preprocessing Pupil Data

Raw pupil data was extracted using the EyeLink Data Viewer software (SR Research, 2020). Pupil data was preprocessed following the guidelines from Mathôt and Vilotijević (2023) and using custom R code incorporating the *GazeR* R package (Geller et al. 2020). Trial‐level blink artifacts were identified and extended by 100 ms before and after each blink, with extended blinks linearly interpolated to maintain continuous pupil fluctuations and to account for missing data occurring during blinking. A low‐pass filter was applied to smooth the interpolated pupil data using the signal R package (Signal Developers, 2023). Trials and participants with substantial missing data (greater than 20%) or anomalous spikes in pupil dilation (likely due to blink interpolation failures) were excluded, resulting in the removal of 7.8% of the data. Trial‐level pupil dilation was then baseline‐corrected by subtracting the average pupil size 400 ms before scene presentation (during the luminance mask presentation) from the pupil size throughout the test period^2^. This approach ensured that changes in pupil size were independent of baseline pupil size and comparable across participants and conditions. Lastly, raw pupil sizes were downsampled from 1000 to 20 Hz for subsequent analyses.

### 2.7 Pupil Dilation Analyses

The primary goal of the current investigation was to assess whether pupil dilation indexes intentional forgetting during retrieval. To this end, we focused only on old scenes and contrasted pupil fluctuation at test between R‐cued old scenes and F‐cued old scenes in incorrect trials (i.e., when participants failed to recognize an old scene). In both cases, participants failed to correctly evaluate the status of the presented scene, suggesting forgetting has occurred either due to failure of remembering (R‐cued miss) or successful intentional forgetting (F‐cued miss). If differential pupil fluctuations at test emerge between R‐cued and F‐cued miss trials, it would suggest that pupil sizes are modulated by forgetting during encoding and thus index the *success* of intentional forgetting. Furthermore, we examined when intentional forgetting was not successful by comparing pupil fluctuations in hit trials between F‐cued scenes subsequently remembered (i.e., unsuccessful intentional forgetting) and R‐cued scenes subsequently remembered (i.e., successful remembering). If differential pupil dilation at test reflects the successful intentional forgetting observed on miss trials, the *failure* of intentional forgetting thus should not modulate pupil dilation. That is, there should be no pupil size differences in hit trials between R and F conditions. To summarize, we predicted different pupil sizes during incidental and intentional forgetting, whereas remembering, whether intentional or incidental, should relate to similar pupil sizes.

Conditional comparison of R‐cued and F‐cued scenes for hit and miss trials was done separately with a time‐series analysis followed by a non‐parametric cluster‐based permutation test, a procedure commonly used in pupillometric research (Albi and Pajkossy 2025; Dandekar et al. 2012; Kloosterman et al. 2015; Privitera et al. 2014). Specifically, the 3‐s test time window was divided into 150 20‐ms time bins. A mixed effect model, with fixed effects of *Cue* (R vs. F) was fit to baseline‐corrected pupil sizes individually for every 20 ms time bin. A significant cluster was identified, and the sum of its statistics was calculated if there were at least 10 consecutive time bins^3^ (i.e., 200 ms) that were below the significance threshold (*alpha* = 0.05). Correction for multiple significance tests was done by a cluster‐based permutation test (Bullmore et al. 1999; Maris and Oostenveld 2007), which involved randomly shuffling the trial‐level conditional label 1000 times. For each iteration, we conducted a time‐series analysis on trial‐level pupil data using shuffled trial labels. We then identified the most significant cluster in the permuted dataset and computed its sum of statistics. After 1000 iterations, a permuted distribution of false‐alarm sums of statistics of permuted clusters was obtained, to which the sum of statistics of the actual cluster was compared. The *p* value for the actual cluster was the proportion of false‐alarm clusters equal to or larger than the true sum of statistics. A significant permuted *p* value indicates that one is unlikely to observe the cluster assuming the null hypothesis is true.

## 3 Results

### 3.1 Behavioral Accuracy

**FIGURE 2:**
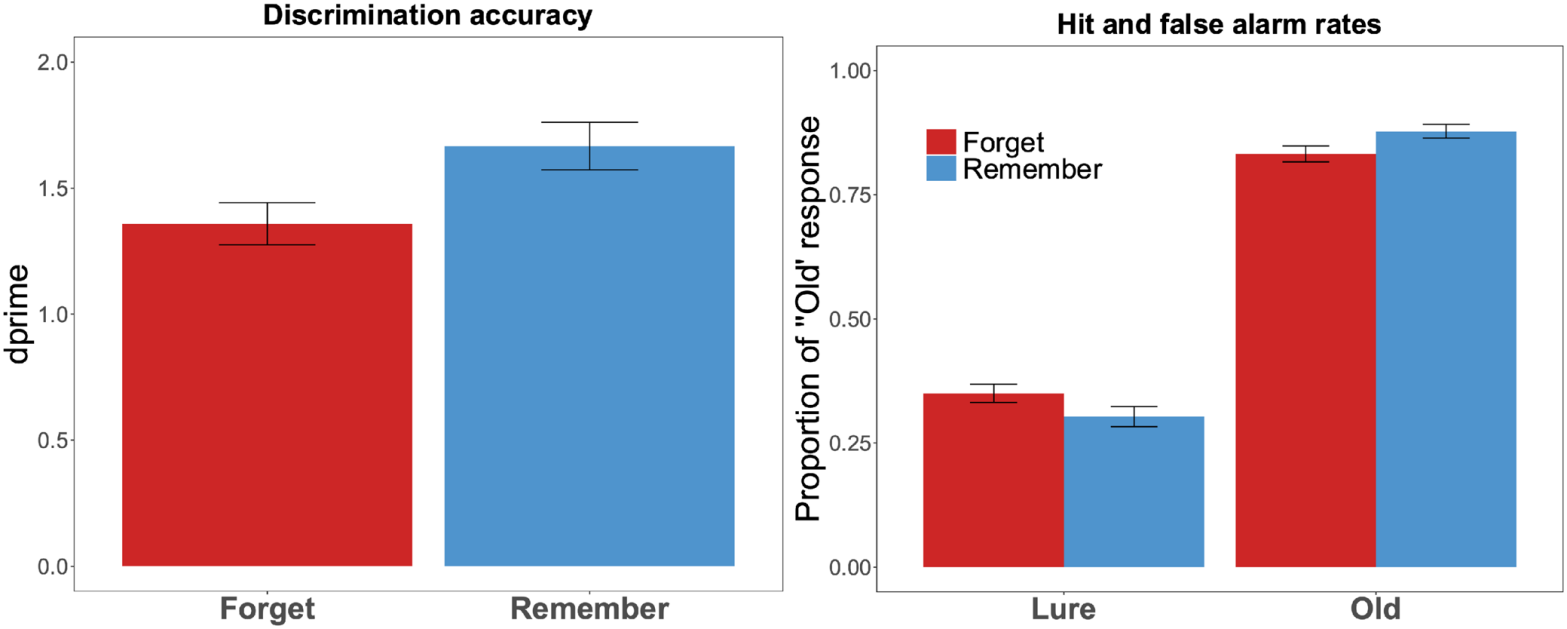
Experiment 1 Hit and False Alarm Rates and Recognition Accuracy (d'). Experiment 1 discrimination accuracy (left panel) and hits and false alarm rates (right panel) as a function of memory cue. Error bars represent standard error of the mean.

Recognition accuracy (expressed as *d*' scores) as well as the hit rates and false alarm rates as a function of memory cue are visualized in Figure 2. A mixed effect regression model with a probit link was fit to trial‐level recognition responses, with *Cue* (R vs. F) and *Type* (old vs. lure) as fixed effects, random intercepts for *Participants* and *Items*, and by‐subject and by‐item random slopes for *Cue* and *Type*. There was a significant fixed effect of *Type*, suggesting that participants were better at correctly judging the Old scene than the Lure scene (*β* = 1.51, SE = 0.13, *z =* 11.30, *p* < 0.001). We also found a significant fixed effect of *Cue* (*β* = 0.20, SE = 0.07, *z =* 2.75, *p* = 0.006). Importantly, there was a significant *Cue* x *Type* interaction (*β* = 0.49, SE = 0.13, *z =* 3.89, *p* < 0.001). Follow‐up analyses to the interaction indicated that participants were better at correctly judging a R‐cued than F‐cued old scenes (*β* = 0.29, SE = 0.10, *z =* 2.87, *p* = 0.004) and better at correctly rejecting R‐cued than F‐cued lure scenes (*β* = 0.20, SE = 0.08, *z =* 2.75, *p* = 0.006). Thus, there was a higher recognition accuracy for R‐cued than F‐cued scenes (*t*(89) = 3.78, *p* < 0.001, *Cohen's d* = 0.40), confirming an item‐method DF effect with natural scenes. Lastly, participants tended to respond liberally for both R (*M* = −0.28, SD = 0.33) and F conditions (*M* = −0.29, SD = 0.29), but the response bias (*c*) did not significantly differ as a function of memory cues, *t*(89) = 0.37, *p* = 0.714, *d*_*z*_ = 0.04.

**FIGURE 3:**
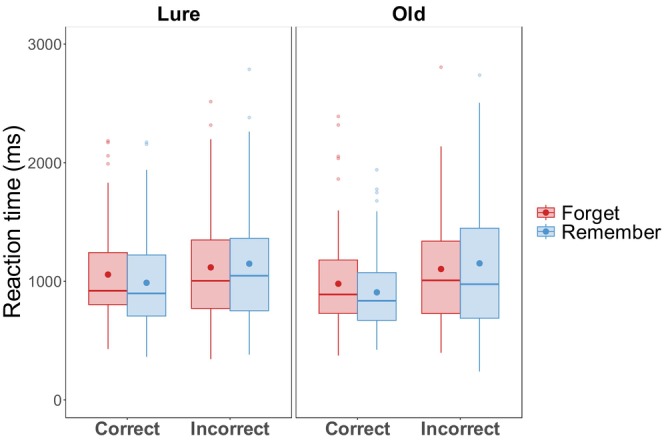
Experiment 1 reaction time. Reaction time (ms) in Experiment 1 as a function of memory cue, accuracy, and image type. Dots represent means in their color‐coded conditions. Horizontal lines represent conditional medians.

A mixed effect model was fit to trial‐level log‐transformed reaction time with *Cue* (R vs. F), *Accuracy* (Correct vs. Incorrect), and *Image Type* (Old vs. Lure) as fixed effects, and random intercepts for *Participants* and *Items*, and by‐subject and by‐item random slopes for *Cue*. Raw reaction time data is visualized in Figure 3. The model yielded a significant fixed effect of *Image Type*, where participants took longer to respond to Lure scene images than Old scene images (*β* = 0.12, SE = 0.03, *t =* 3.96, *p* < 0.001). There was also a significant fixed effect of *Cue*, where participants took longer when responding to F‐cued than R‐cued scenes (*β* = 0.07, SE = 0.03, *t =* 2.30, *p* = 0.022). Finally, we obtained a significant *Image Type* × *Accuracy* interaction (*β* = 0.20, SE = 0.06, *t =* 3.26, *p* = 0.001), driven by faster correct responses for Old scenes than Lure scenes (*β* = 0.11, SE = 0.02, *z* = 5.54, *p* < 0.001), whereas the incorrect responses for Old and Lure scenes did not differ in reaction time (*β* = 0.07, SE = 0.04, *z* = 1.80, *p* = 0.072). All other fixed effects and interactions were not significant (all *ps* > 0.304).

### 3.2 Intentional Forgetting Modulates Pupil Fluctuations

**FIGURE 4:**
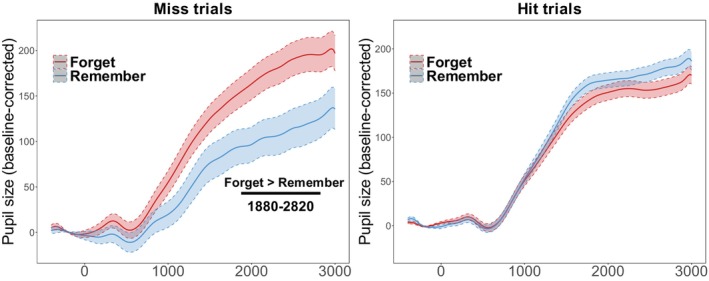
Experiment 1 pupil fluctuation at test. Conditional means of pupil fluctuations are plotted, contrasting intentional (F‐cued) and incidental (R‐cued) forgetting for miss (left) and hit (right) trials. The onset of the test image was denoted by 0. The error ribbons represent SEM. Significant time bins (all *p* < 0.05) were indicated by the black line.

To investigate if intentional forgetting modulates pupil dilation at test, a time‐series analysis was performed individually on hit and miss trials for all 150 20‐ms time bins as a function of Cue. The baselined pupil dilation data for hit and miss trials are visualized in Figure 4. For each time bin, a mixed effect model was fit to baseline‐corrected pupil size with a fixed effect of *Cue* (R vs. F), random intercepts for *Participants* and *Items*, and by‐item random slopes for *Cue*. We found a significant cluster between 1880 and 2820 ms after the onset of the test scene image, where there was a greater pupil dilation for F‐cued trials than R‐cued trials, corrected by the cluster‐based permutation test (*p* = 0.041). This suggests that intentional forgetting is associated with greater pupil dilation than incidental forgetting at approximately the last second of the test scene presentation. The time‐series analyses on pupil dilation at test in hit trials, however, did not identify a significant cluster (all *ps* > 0.163), suggesting that when intentional forgetting failed—that is, the undesirable outcome when F‐cued scenes were nevertheless remembered—pupil size did not significantly differentiate unsuccessful intentional forgetting from successful remembering.

## 4 Discussion

We obtained a robust item‐method DF effect with natural scenes. Critically, we found that the pupil dilates more during intentional forgetting compared to incidental forgetting, evident in increased pupil dilation for subsequently forgotten scenes that were F‐cued compared to forgotten scenes that were R‐cued. We only observed this pattern when Old scenes were subsequently forgotten. Importantly, pupil dilation on correct trials (hits) was similar for R‐cued and F‐cued scenes. This suggests that pupil dilation only indexed the *success* of intentional forgetting at the time of retrieval.

Greater pupil dilation during long‐term memory retrieval of old items compared to pupil dilation in response to new items was initially explained by the retrieval effort account, offered by Võ et al. (2008), according to which pupil at test reflects the cognitive effort required to successfully retrieve studied items. One might consider whether the obtained pattern is due to an increased effort to retrieve F‐cued compared to forgotten R‐cued forgotten scenes. Our reaction time results do not support this notion. We did observe longer reaction time when judging lure scenes than old scenes, as rejecting lures is typically more difficult and takes more effort than rejecting old scenes. Importantly, reaction times did not differ between scenes that were intentionally versus incidentally forgotten. This suggests that the cognitive effort involved in arriving at an incorrect judgment was comparable across the two conditions, which is inconsistent with the mental effort explanation.

Alternatively, one might suggest that the observed result arises because of the memory strength difference between R‐cued and F‐cued scenes. Indeed, previous studies have found pupil dilation correlates with memory strength (Otero et al. 2011, but see Gross and Dobbins 2021) (e.g., Kafkas and Montaldi 2011; Otero et al. 2011; Taikh and Bodner 2022). Specifically, Taikh and Bodner (2022) varied memory strength by instructing some words to be encoded deeply versus others processed shallowly. Their results indicated that pupil dilation increased when shallowly‐encoded items were incorrectly endorsed as new items compared to when deeply‐encoded items were incorrectly endorsed as new items (although they did not specifically test this comparison in their investigation). However, the memory strength differences examined in previous pupillometric research were achieved by upregulating encoding strength (e.g., through deeper and more elaborative encoding). In contrast, the forget instruction in the present study downregulated memory representation via active mechanisms such as inhibition or suppression. While both manipulations produce quantitatively similar differences in memory strength, the underlying mechanisms could be distinct. As a result, there may be reasons to suspect that the findings of increased pupil dilation associated with weaker items in prior studies may not generalize to F‐cued items in our study. To directly test this explanation, in Experiment 2, we experimentally manipulated memory strength with a similar experimental design as in Experiment 1.

## 5 Experiment 2

Experiment 2 was designed to test if greater pupil dilation for intentionally forgotten scenes was purely due to memory strength differences, or whether it was due to the differences arising from processes aimed at downregulating memory in response to F‐cues. The selective rehearsal account posits that the DF effect is due to the elaborative rehearsal of R‐cued items, with no active processes acting upon F‐cued items. Therefore, the emergence of the DF effect at retrieval is due to more strongly encoded R‐cued items compared to relatively weakly encoded F‐cued items. If selective rehearsal is the only mechanism responsible for the DF effect and if larger pupil dilation in the intentional forgetting condition is due to memory strength difference as a result of rehearsal termination, we would expect to observe similar pupillometric patterns if we experimentally strengthened some items. That is, we expect to see incorrectly judging weakly encoded Old items (analogous to F‐cued scenes in Exp 1) to elicit greater pupil dilation than strongly encoded Old items (analogous to R‐cued scenes in Exp 1) at test. We directly tested this prediction in Experiment 2 by eliminating R/F cues and instead pre‐exposing a subset of scenes prior to the encoding phase so that, at the end of the encoding phase, scenes were either presented twice versus once to create a difference in memory strength.

## 6 Method

### 6.1 Participants

Participants were 53 undergraduate students who participated in exchange for course credit from the same university as in Experiment 1. No participants recruited in Experiment 2 participated in Experiment 1.

### 6.2 Stimuli

The complete stimuli set consisted of 80 natural scenes with the 40 natural scenes we used in Experiment 1 and 40 additional scenes selected from the same dataset (FIGRIM; Bylinskii et al. 2015). We doubled the number of stimuli to prevent a ceiling effect for non‐strengthened scenes to ensure a strengthening effect between strengthened and non‐strengthened conditions. The additional 40 scenes were again ensured to be largely asymmetrical and to not contain humans. Two scramble box images were created for each natural scene for luminance masks. All scenes were set to a resolution of 800 × 600.

### 6.3 Procedure

Participants completed a preview phase, a study phase, and a test phase. During the preview phase, participants studied 40 natural scenes. Each preview trial began with an initial fixation cross presented for 1 s, followed by a luminance mask for 2 s, and followed by a scene image for 3 s. Participants were told that scenes they learned in the preview phase would appear in the subsequent memory test. Immediately after, participants completed a study phase where they studied 80 natural scenes, half of which had already been presented in the preview phase (Previewed scenes) and half of which they studied for the first time (Non‐Previewed scenes). Unlike in Experiment 1, no memory cues were given, and participants were instructed to remember all the scenes for the subsequent memory test. At test, participants were presented with either a Previewed scene (strong encoding) or a Non‐Previewed scene (weak encoding) in either its original version (Old) or its mirrored variant (Lure), to which they needed to judge the status of the presented image as either Old or Lure. The timing parameters of the study and test phases of Experiment 2 are the same as in Experiment 1. The assignment of scenes to Old and Lure conditions was counterbalanced across participants. The same preprocessing procedure was employed to remove incomplete or missing pupil data (10.1% of data removed).

### 6.4 Analytic Plan

The main focus of Experiment 2 was to rule out the memory strength explanation of the results demonstrating greater pupil dilation when scenes were intentionally forgotten, observed in Experiment 1. The primary analysis centered around contrasting pupil fluctuation at test between weakly encoded (non‐previewed) and strongly encoded (previewed) scenes in hit and miss trials. If the difference in pupil dilation between intentional and incidental forgetting can be explained by their memory strength difference, weakly and strongly encoded scenes that were incorrectly evaluated should resemble incorrectly evaluated F‐cued and R‐cued scenes in Experiment 1 and thus produce similar pupillometric findings. That is, we should observe, in miss trials, a greater pupil dilation for weakly encoded scenes than strongly encoded scenes. Statistical analyses on conditional comparison followed the procedure in Experiment 1.

## 7 Results

### 7.1 Behavioral Accuracy

**FIGURE 5:**
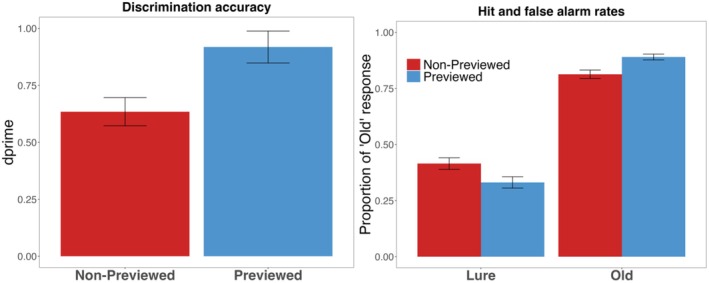
Experiment 2 hit and false alarm rates and recognition accuracy. Behavioral accuracy in Experiment 2 showing recognition accuracy (left) and hits and false alarm rates (right) as a function of Preview (Previewed and Non‐Previewed). Error bars represent standard errors.

A mixed regression model with a probit link was fit to trial‐level behavior response with *Preview* (Previewed vs. Non‐Previewed) and *Type* (Old vs. Lure) as fixed effects, random intercepts for *Participants* and *Items, and* by‐subject and by‐item random slopes *for Preview* and *Type*. The results of recognition accuracy expressed in *d'* scores and hit rates and false alarm rates are visualized in Figure 5. We found a greater likelihood of judging “old” to old than lure scenes (*β* = 1.28, SE = 0.12, *z =* 10.24, *p* < 0.001). We also found that participants were more likely to provide “old” responses for non‐previewed than previewed scenes (*β* = 0.28, SE = 0.08, *z =* 3.56, *p* < 0.001). Finally, there was a significant *Preview* x *Type* interaction (*β* = 0.62, SE = 0.11, *z =* 5.67, *p* < 0.001), where follow‐up analyses indicated that there were greater hit rates (*β* = 0.34, SE = 0.08, *z =* 4.25, *p* < 0.001) and lower false alarm rates for previewed than non‐previewed scenes (*β* = 0.24, SE = 0.07, *z =* 3.55, *p* < 0.001).Thus, the strengthening manipulation enhanced correct responses and reduced errors. Indeed, an independent t‐test performed on subject‐level recognition accuracy (*d'*) yielded significantly higher accuracy for previewed than non‐previewed scenes, *t*(52) = 4.01, *p* < 0.001, *d*_*z*_ = 0.55. Lastly, participants tended to respond liberally for both previewed (*M* = −0.38, SD = 0.30) and non‐previewed conditions (*M* = −0.35, SD = 0.35), but the response bias (*c*) did not significantly differ as a function of preview, *t*(50) = 0.58, *p* = 0.563, *d*_*z*_ = 0.08. Overall, behavioral results confirmed the effectiveness of the strengthening manipulation.

**FIGURE 6:**
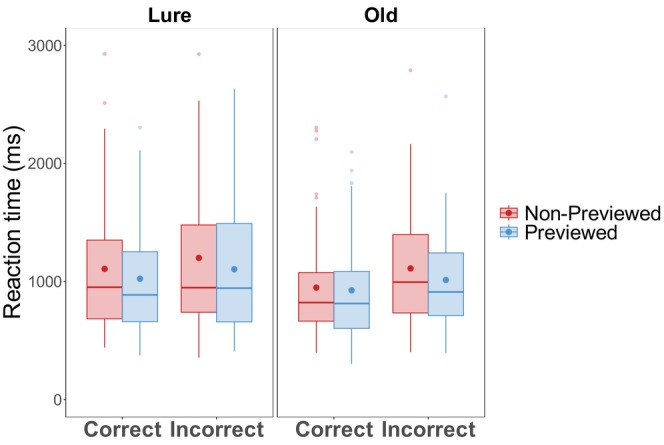
Experiment 2 reaction time. Reaction time in Experiment 2 as a function of Preview (Previewed vs. Non‐Previewed), Accuracy (Correct vs. Incorrect), and Image Type (Old vs. Lure). Dots represent subject‐level means in their color‐coded conditions. Horizontal lines represent conditional medians.

A mixed effect regression model was fit to log‐transformed reaction time in Exp 2 with *Preview* (Preview vs. Non‐Previewed), *Accuracy* (Correct vs. Incorrect), and *Image Type* (Old vs. Lure) as fixed effects, random intercepts for *Participants* and *Items*, and by‐subject and by‐item random slopes for *Preview* and *Image Type*. Raw reaction time is visualized in Figure 6. The omnibus model yielded a significant fixed effect of *Accuracy*, where participants were faster to make correct than incorrect responses (*β* = 0.24, SE = 0.04, *t =* 5.46, *p* < 0.001). We also found a significant *Image Type* × *Accuracy* interaction (*β* = 0.24, SE = 0.06, *t =* 4.33, *p* < 0.001), where incorrect responses to Old scenes were faster than those to Lure scenes (*β* = 0.11, SE = 0.04, *z =* 2.87, *p* = 0.004) whereas participants correctly judged Lure scenes slower than Old scenes (*β* = 0.12, SE = 0.02, *z =* 5.33, *p* < 0.001), collapsing across *Preview* conditions. All other fixed effects, two‐way, and three‐way interactions did not emerge as significant (all *ps* > 0.090). Importantly, there were no fixed effects or interactions involving *Preview*, neither on correct nor on incorrect trials.

### 7.2 Memory Strength (Per Se) Does Not Modulate Pupil Dilation

**FIGURE 7:**
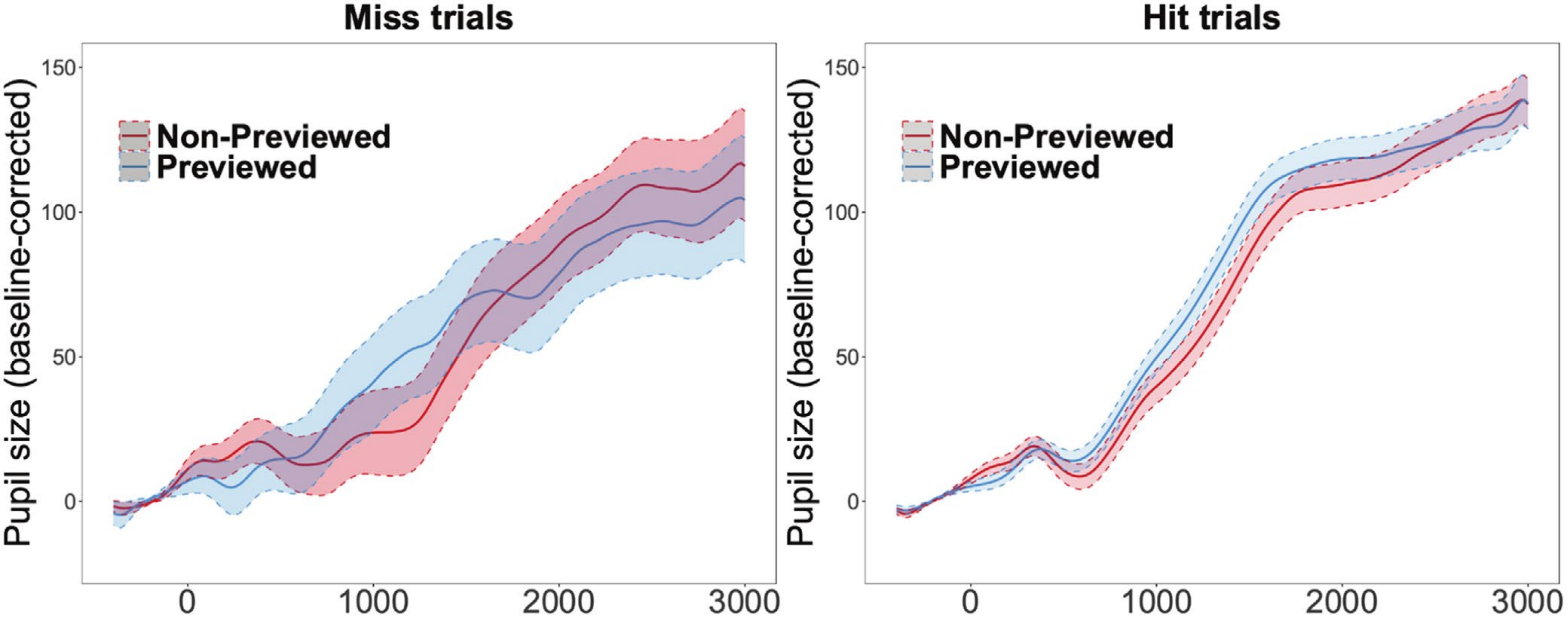
Experiment 2 pupil fluctuation at test. Experiment 2 conditional means of pupil fluctuations during test scene image presentation are plotted. Pupil dilation when participants made a miss (left) and hit (right) to weakly encoded (non‐previewed) and strongly encoded (previewed). Significant time bins (all *p* < 0.05) were indicated by the black line. The onset of the scene image was denoted by 0. The error ribbons represent SEM.

To examine if strengthened memory would modulate pupil dilation at test, a time‐series significance test was performed individually on hit and miss trials for all 150 20 ms time bins as a function of *Preview* (Previewed vs. Non‐previewed). The baseline‐corrected pupil dilations are visualized in Figure 7. For each time bin, a mixed effect model was fit to baseline‐corrected pupil size with a fixed effect of *Preview* (Previewed vs. Non‐previewed), random intercepts for *Participants* and *Items*, and by‐subject random slopes for *Preview*. When participants correctly judged the status of the presented scene image, there was no significant cluster found (all *ps* > 0.089). Critically, we did not find pupil size differences between previewed and non‐previewed conditions across time when participants made incorrect judgments to Old scenes (all *ps* > 0.142) as a function of preview condition. This finding is not consistent with the explanation that differential memory strength drives the modulated pupil dilation at test. Note that there was a comparable number of data points going in the analyses for Experiment 1 (256 data points) and Experiment 2 (286 data points) on miss trials, and therefore the null finding in Experiment 2 is not due to insufficient power to detect the effect.

## 8 Discussion

In Experiment 2, where some scenes were strengthened by previewing, weakly encoded old scenes did not differ in pupil dilation from strongly encoded old scenes when participants made incorrect judgments. This finding is in contrast with the pattern observed in Experiment 1 and does not support the explanation that greater pupil size when scenes were intentionally forgotten was merely the result of memory strength differences between R‐cued and F‐cued items. From a theoretical standpoint, it also implies that additional processes must have been invoked upon receiving the forget cue. If the forget cue only induced a termination of rehearsal, F‐cued scenes would then be treated similarly to the non‐previewed scenes in relation to R‐cued and previewed scenes, which we did not observe any differential pupil fluctuation when judged incorrectly. The distinct pupillometric modulation occurring only when scenes were intentionally forgotten but not when scenes were weakly encoded suggests that pupil size changes at test reflect unique processes associated with intentional forgetting, likely involving the recruitment of inhibitory mechanisms that suppress representations followed by F cues or context unbinding mechanisms.

## 9 General Discussion

The LC‐NE system, a key regulator of arousal and cognitive control, forms a functional triad with the prefrontal cortex and hippocampus, both of which are related to intentional forgetting. The activity of the LC‐NE system is closely associated with pupil size fluctuations. The current investigation examined whether pupil dilation during retrieval would signal successful intentional forgetting processes. Using an item‐method DF paradigm with complex natural scenes in Experiment 1, we found a greater pupil dilation when scenes were *successfully* intentionally/actively forgotten than when scenes were incidentally/passively forgotten. In both cases, participants made an inaccurate judgment in evaluating the type of tested image but differed in terms of the intention to either forget or remember, and therefore, the difference in pupil size between the two conditions likely reflected different underlying mechanisms. On the contrary, when intentional forgetting failed (i.e., F‐cued scenes were nonetheless correctly evaluated), we did not observe any pupillometric differences. Failure of intentional forgetting elicits nearly identical pupil dilation at test as successful remembering. To our knowledge, this is the first study that has examined pupillometry with intentional forgetting during retrieval and revealed a unique pupillometric signature related to *successful* intentional forgetting.

Based on existing memory‐related pupillometric findings, one can argue that the increased pupil dilation for subsequently forgotten F‐cued scenes may have several possible explanations. First, it is possible that F‐cued scenes have relatively weaker memory representations compared to R‐cued scenes, and thus require greater cognitive effort to retrieve, even though participants ultimately failed to do so. Indeed, many studies found that pupil fluctuation is a physiological marker of increased voluntary effort during memory retrieval (Goldinger and Papesh 2012; Papesh et al. 2012; Võ et al. 2008). The reaction time data in Experiment 1 provided evidence to suggest otherwise. We observed that participants were slower in responding to Lure scenes than Old scenes because of the difficulty of matching highly perceptually similar lures to their stored memory representation of the original versions, suggesting that effortful retrieval indeed accompanies slower judgment time. Critically, we did not observe a reaction time difference concerning the critical comparison between the F‐cued and R‐cued miss responses. This suggests that incorrectly evaluating F‐cued scenes is not more difficult than incorrectly evaluating R‐cued scenes, which poses difficulty for the cognitive effort explanation of our findings.

The memory strength difference between intentional and incidental forgetting conditions also cannot adequately explain the observed effect. Whether pupil sizes reflect differential encoding strength remains inconclusive in the literature. While some studies have found stronger memory is related to greater pupil dilation than weaker memory (e.g., Ariel and Castel 2014; Heaver and Hutton 2011; Taikh and Bodner 2022; Papesh et al. 2012; Otero et al. 2011), others have found that pupil size does not reflect the veridical strength of the items through Level‐of‐Processing (LOP) manipulation (e.g., Gross and Dobbins 2021; Siefert et al. 2024). Moreover, quantitatively similar strength differences can be achieved through various empirical manipulations (i.e., repetition, LOP manipulation, prolonged study time, etc.), which can vary in their underlying mechanisms and influence different components of episodic memory traces (i.e., Malmberg and Shiffrin 2005). F‐cued items and other weakly encoded items, despite their diminished performance level compared to R‐cued and other strongly encoded counterparts, may be expressed differentially in pupil dilations at test because different underlying mechanisms may be responsible for the memory performance level differences. We directly examined this notion in Experiment 2 by using an identical procedure as in Experiment 1, except scenes were strengthened through repetition to create conditions that were strength‐wise analogous to R‐cued and F‐cued scenes. We did not find a significant difference in pupil dilation between weakly and strongly encoded scenes when they were incorrectly endorsed. This finding suggests that F‐cued items in the present study were not qualitatively analogous to weakly encoded items reported in prior research. Instead, pupil dilation reflects processes unique to the lingering effects of intentional forgetting during retrieval, rather than merely indexing memory strength.

Our results are consistent with the prediction based on the mismatch literature, where pupil size changes index the magnitude of mismatch between the presented visual input and the stored memory representation of intentionally forgotten scenes. Specifically, we propose that mismatch arises from (1) the less complete memory traces of F‐cued scenes due to inhibition and/or (2) the mismatch in encoding and retrieval context because of the item‐context separation induced by forget cues. Generally, semantic mismatch has been observed with increased pupil dilation. Kuipers and Thierry (2011) presented English‐speaking adults with highly familiar object and animal pictures, along with either a match (the name of the object) or a mismatch verbal label (unrelated word). They found an increased pupil dilation for mismatch trials than for match trials, a finding similarly observed by Renner and Włodarczak (2017). Importantly, the pupillary response to input‐representation mismatch was also found in episodic memory retrieval. Siefert et al. (2024, experiment 1) demonstrated that pupillary responses are sensitive to input‐representation mismatches. In their study, participants encoded words and pictures during the study phase and were later tested with a Yes/No recognition task using words (e.g., studying a picture of a lamp and being tested with the word “lamp”). Participants were randomly assigned to either shallow processing (e.g., determining whether the item was a word) or deep processing (e.g., judging whether the item was organic) conditions. Notably, pupil dilation did not vary as a function of the level‐of‐processing manipulation. Instead, they found that the mismatch condition elicited greater pupil dilation than the match condition. Consistently, the mismatch between the memory representation and the visual input is greater when the visual input is compared to the less complete and inhibited representation of F‐cued scenes, leading to increased pupillary responses.

Beyond item representation mismatch, discrepancies in contextual information may also contribute to the observed pupillometric effect. Chiu et al. (2021) used a neural classifier to index item and contextual information in an item‐method DF paradigm. Their findings indicated that forget cues led to the unbinding of the item and its context, evident in a downregulation of item information along with an upregulation of contextual information. The trial‐level degree of unbinding during encoding was also correlated with the magnitude of the DF effect. Based on this finding, the increased pupil dilation for intentional forgetting may also reflect contextual mismatch, which is greater for F‐cued scenes due to the separation of context induced by the forget cue. Collectively, the increased pupil dilation observed for intentionally forgotten items may reflect the representation‐input mismatch on both item representation and contextual levels.

Experiments 1 and 2 together also provide insights into the theoretical explanation of the DF effect. The selective rehearsal account of the DF effect posits that the reduced memory accuracy for F‐cued items is due to a termination of rehearsal, compared to continued rehearsal of R‐cued items (e.g., Basden et al. 1993; Bjork 1970; MacLeod 1999). If selective rehearsal is the only difference in cognitive processes participants had upon receiving R and F cues, the memory strength difference between R‐cued and F‐cued items should be analogous to strengthened and non‐strengthened items. The clear dichotomy of pupillary patterns in miss trials we observed in Experiments 1 and 2, however, suggests that additional processes must have occurred during memory cues that result in the unique pupillometric signature of intentional forgetting in Experiment 1, including active recruitment of suppression mechanisms and/or unbinding of items from their episodic context.

Prior to our study, only two studies have investigated pupil dilation related to directed forgetting (Lee 2018; Scholz and Dutke 2019). Both studies employed a modified item‐method DF paradigm with an additional baseline condition. In addition to instructions to remember or forget items, participants were instructed to either ignore (Scholz and Dutke 2019) or do nothing with the item (Lee 2018) upon receiving a baseline cue. Both studies reported greater pupil dilation in response to remember cues compared to forget cues, with the latter showing no significant difference from the baseline condition. However, neither study found significant behavioral differences between the forget and baseline conditions. This raises the possibility that participants treated the baseline condition similar to the forget condition or that the forget instructions were less effective with the inclusion of baseline cues, leading to reduced engagement of active cognitive processes and thus not producing any pupillometric effects. Therefore, no definitive conclusions can be drawn from the prior evidence that pupil dilation *does not* reflect unique processes upon receiving the forget cue. While replicating their pupillometric findings during the memory cue period^4^, our investigation provided novel evidence that pupil dilation signals the successful intentional forgetting at the time of test, suggesting that the DF effect is driven not only by reduced representation at encoding but also by impaired *retrieval* (Chiu et al. 2021; Rummel et al. 2016; Marevic and Rummel 2020; Marevic et al. 2018).

In summary, the present results demonstrate that pupil dilations at test reflect the successful outcome of intentional forgetting, which is distinct from incidental forgetting. This effect is not driven by memory strength differences, as readily ruled out by Experiment 2. Instead, the modulated pupil dilation related to intentional forgetting may reflect the lingering inhibition induced by forget cues, indexed for impaired retrieval processes, or a greater item and contextual mismatch between memory representation and visual inputs as a result of intentional forgetting.

## Author Contributions

**Huiyu Ding:** writing – original draft, visualization, methodology, conceptualization, investigation, software, data curation, formal analysis. **Jonathon Whitlock:** validation, formal analysis, software, methodology. **Lili Sahakyan:** writing – review and editing, supervision, conceptualization, investigation.

## Conflicts of Interest

The authors declare no conflicts of interest.

## Supporting information


**Data S1:** psyp70119‐sup‐0001‐DataS1.docx.

## Data Availability

The data that support the findings of this study are openly available in Pupil Fluctuations Signal Active Forgetting of Natrual Scene at https://osf.io/46hxm/files/osfstorage.

## Endnotes

^1^
We initially collected 50 participants in Experiment 1 but subsequently discovered a counterbalancing issue with our code where some images were consistently given the same memory instruction across conditions. To remedy it, we collected the data from 40 additional participants. The results did not change with the second sample collected. The second sample replicated the findings from the first sample, and the original results with 50 participants are reported in the Supplemental section.

^2^
Baseline pupil size did not significantly differ across behavioral accuracy and memory cues.

^3^
This criterion was determined because the latency of pupil light response is around 200 ms, and it is consistent with previous literature on time‐series pupillometric analyses (e.g., Mathot et al. 2017; Hershman et al. 2022).

^4^
Pupil dilation during instruction period in Experiment 1 was reported in Supporting Information.
