# Supplementary material for: Pupil Fluctuations Signal Intentional Forgetting of Natural Scenes
Source: Psychophysiology. 2025 Aug 1;62(8):e70119. doi: 10.1111/psyp.70119 (PMC12317212; doi:10.1111/psyp.70119)

**Supplemental materials**

Figure 8 visualizes the critical comparison on pupil dilation between intentional and incidental forgetting with the original sample size we initially collected in Experiment 1 (N = 50). We observed a similar pattern with the complete sample that we reported, where incorrectly evaluating F-cued old scenes elicited greater pupil dilation than incorrectly evaluating R-cued scenes starting from 880 ms after the onset of the scene and persisted to the offset.

Figure 8.

*Pupil Dilation Data With Initial Sample Size*


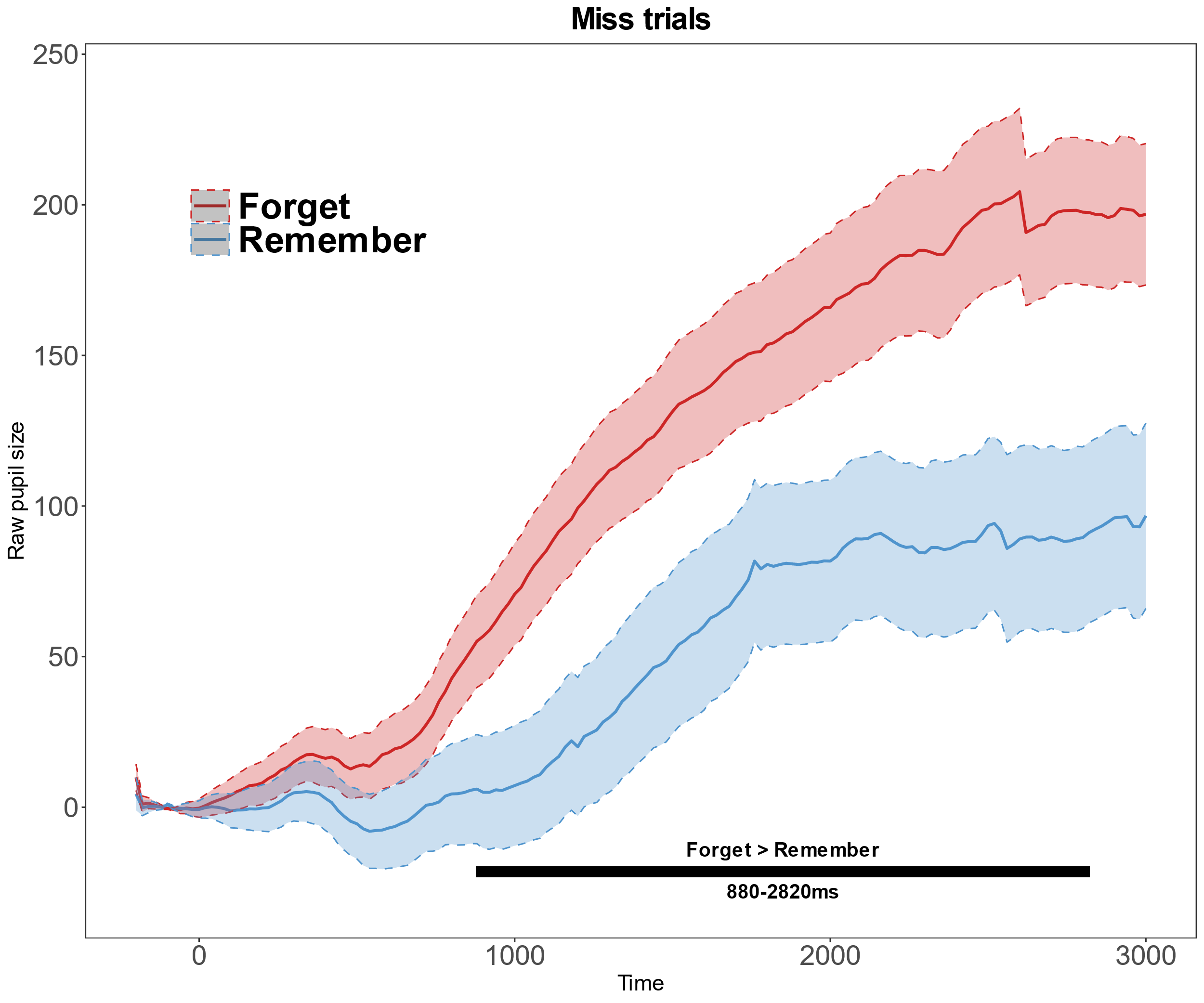


Figure 9 visualizes the pupil dilation during memory instruction period in Experiment 1. R instruction elicited greater pupil dilation than F instruction, emerging 920ms after the onset of memory cue and persisted throughout the cue presentation period.

Figure 9.

*Pupil Dilation During Memory Cues*


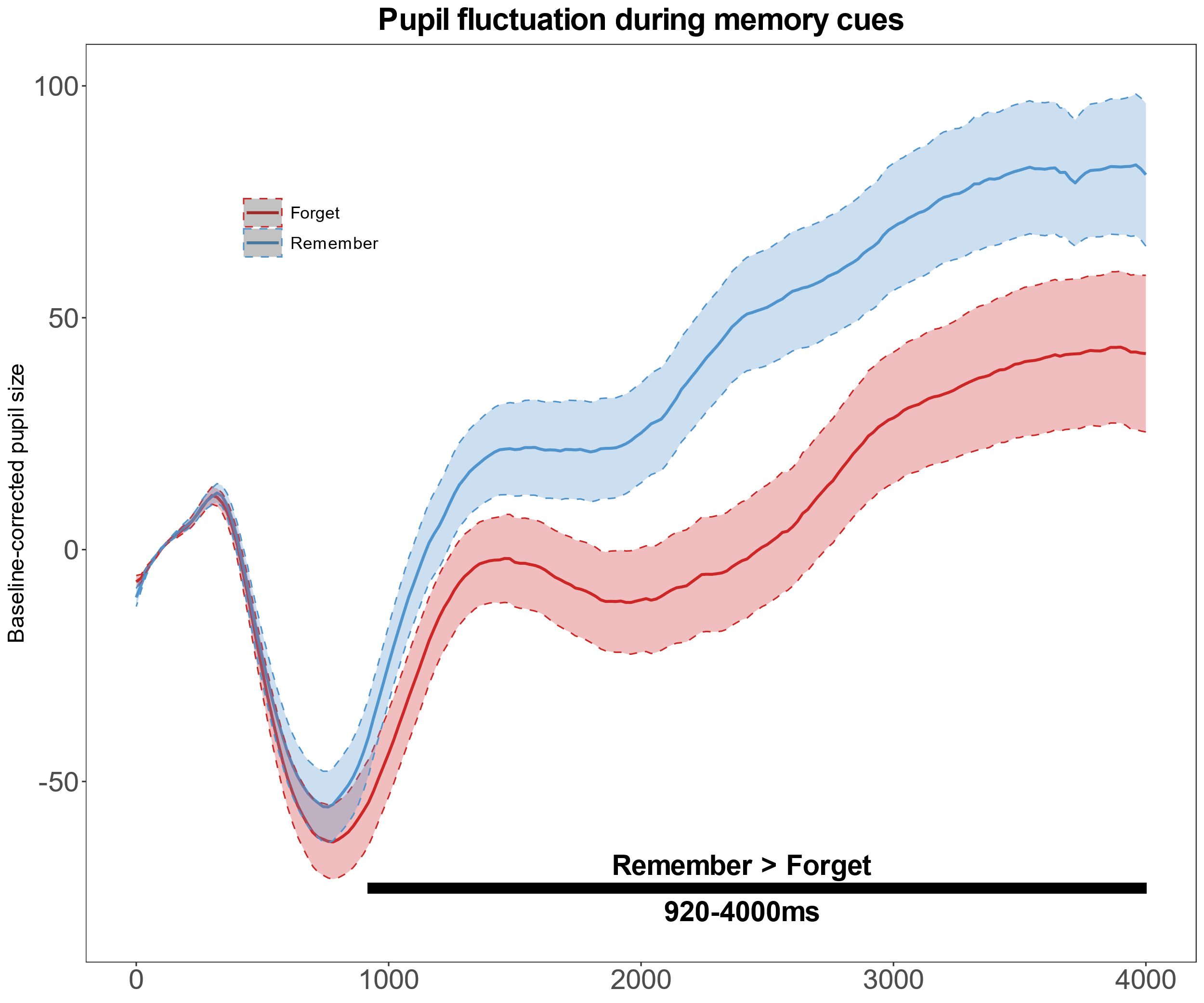

Supplement: Supplementary file 1 — Data S1: psyp70119‐sup‐0001‐DataS1.docx. [file PSYP-62-e70119-s001.docx]
